# Microendoscopic Cervical Anterior Decompression and Fusion: A Technical Note

**DOI:** 10.7759/cureus.91446

**Published:** 2025-09-01

**Authors:** Keiji Nagata, Hiroshi Hashizume, Hiroshi Iwasaki, Masatoshi Teraguchi, Hiroshi Yamada

**Affiliations:** 1 Department of Orthopedic Surgery, Wakayama Medical University, Wakayama, JPN; 2 School of Health and Nursing Science, Wakayama Medical University, Wakayama, JPN

**Keywords:** acdf, airway obstruction, cervical fusion, dysphagia, mecaf, microendoscopic surgery, minimally invasive spine surgery

## Abstract

Microendoscopic cervical anterior decompression and fusion (MECAF) is a novel minimally invasive surgical technique developed to reduce postoperative complications commonly associated with anterior cervical discectomy and fusion (ACDF), such as airway obstruction, dysphagia, and recurrent laryngeal nerve paralysis, particularly in elderly patients and those with comorbidities. Utilizing an 18-mm tubular retractor and a 25-degree endoscope, MECAF minimizes soft tissue trauma by approaching along the lateral border of the omohyoid muscle, enabling safe en bloc retraction of the trachea and esophagus. Under endoscopic visualization, anterior cervical discectomy, cage insertion, and fixation with a self-locking plate are performed. Between 2021 and 2024, 20 patients (mean age 60.0 ± 15.3 years) underwent MECAF for cervical disc herniation, myelopathy, radiculopathy, or myeloradiculopathy. The procedure was initially applied to single-level cases and subsequently extended to two-level surgeries, yielding favorable outcomes. Notably, there were no instances of postoperative dyspnea, hoarseness, or dysphagia. These findings suggest that MECAF is a safe, effective, and less invasive alternative to conventional ACDF.

## Introduction

Cervical anterior fusion is a well-established surgical procedure with several significant advantages over cervical laminoplasty (decompression surgery), including reduced invasiveness to muscles and soft tissues, as well as the ability to effectively correct spinal alignment. However, anterior cervical discectomy and fusion (ACDF) carries a risk of serious complications, notably postoperative acute respiratory and swallowing disorders related to swelling of the posterior pharyngeal space (a soft tissue compartment located behind the pharynx that can easily become edematous after anterior cervical surgery), as well as recurrent laryngeal nerve paralysis. Elderly patients, particularly those aged 65 years or older, are reported to face an elevated risk of respiratory complications and dysphagia. A previous investigation involving patients aged 70 years or older indicated hazard ratios of 2.69 for respiratory complications and 4.96 for dysphagia [1-3]. Moreover, common comorbidities in elderly populations, such as hypertension, diabetes, asthma, obesity, chronic obstructive pulmonary disease (COPD), and upper cervical surgeries involving the C3 vertebral level, have also been identified as significant risk factors for respiratory complications following ACDF [3-6].

Traditionally, the Smith-Robinson approach is widely adopted as the standard method for discectomy and fusion using tricortical bone grafts. In contrast, the Cloward approach employs a circular trephine to remove the disc and a cylindrical bone dowel for fusion. While the Smith-Robinson and Cloward approaches remain standard for ACDF, these methods often present challenges in higher cervical levels due to the increasingly lateral position of the sternocleidomastoid muscle cranially.

To address the limitations of these conventional techniques, particularly the risk of soft tissue swelling due to forceful retraction, we developed a less invasive surgical method utilizing microendoscopic surgery aimed at reducing postoperative airway complications. Recently, endoscopic procedures have gained popularity in spinal surgery, offering the advantage of minimal surgical invasiveness. However, previously reported endoscopic techniques have not been widely adopted, partly because detailed descriptions of the surgical approach are lacking, and their impact on airway complications remains insufficiently documented. Our method utilizes an 18-mm diameter tubular retractor system, combined with endoscopic visualization, to minimize tissue trauma and facilitate safe anterior exposure. In this report, we present our surgical technique of microendoscopic cervical anterior decompression and fusion (MECAF) along with the initial clinical outcomes.

## Technical report

We have developed the following surgical procedure for the treatment of degenerative cervical spine disease.

Intraoperative position

The patient is positioned supine, with a rolled pillow placed beneath the upper scapular region and multiple towels placed beneath the head to slightly elevate it. The lower jaw is gently extended upward, creating a subtle slope. This positioning mimics the sniffing position commonly used for endotracheal intubation, effectively minimizing soft tissue tension during cervical exposure.

Surgical technique for MECAF

Surgical dissection proceeds along the lateral border of the omohyoid muscle in a cranio-caudal direction, retracting the muscle together with the trachea and esophagus as a single unit, providing safe and direct access to the anterior vertebral body. After exposure, the dorsal surface of the longus colli muscle is carefully dissected, revealing the transverse processes bilaterally and allowing visualization of the uncovertebral (Luschka) joints. Disc level confirmation is achieved using a 23-gauge needle, followed by disc staining with dye to enhance visualization.

**Figure 1:**
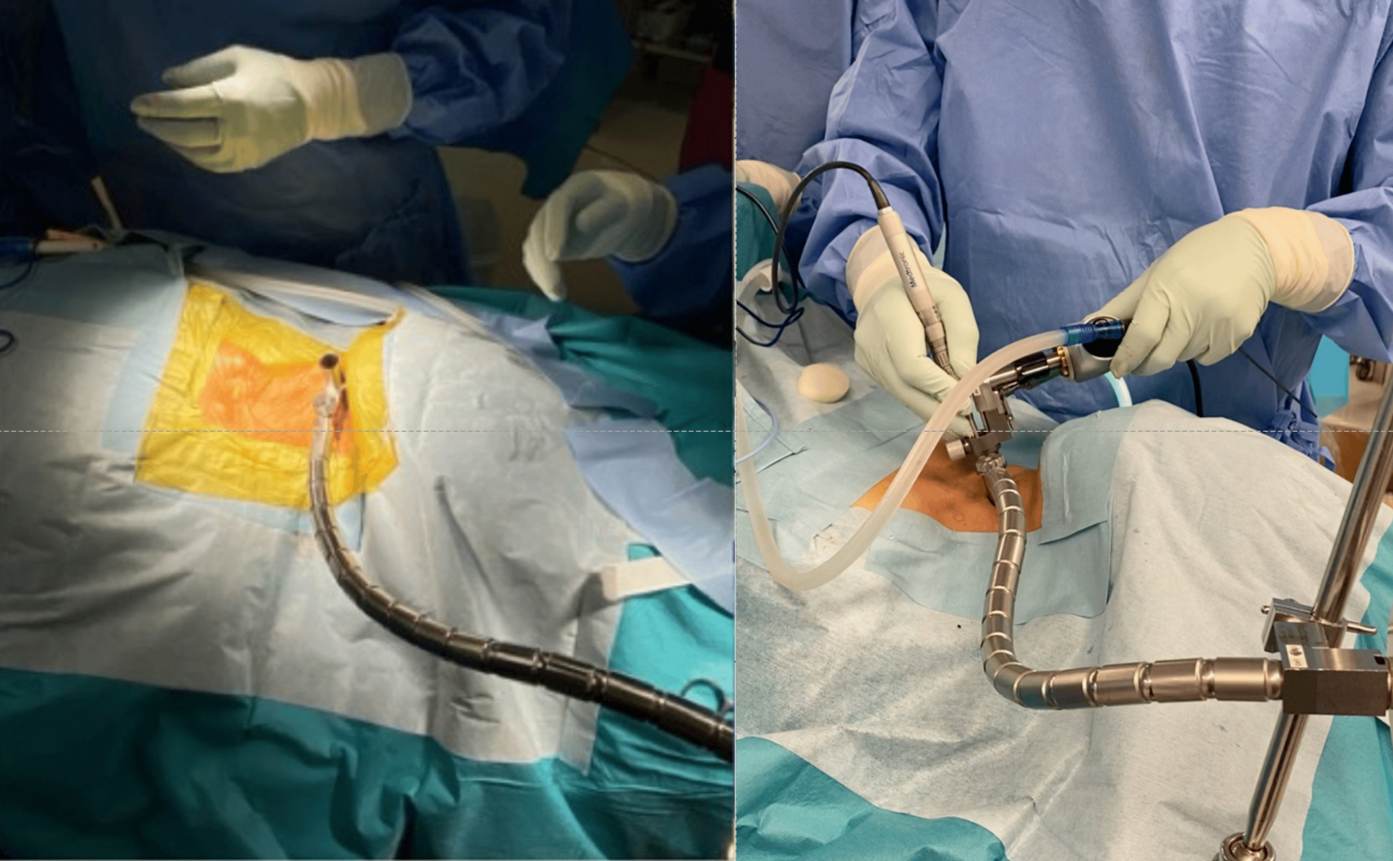
Intraoperative image Placement of an 18-mm tubular retractor over the intervertebral disc space, stabilized with a mechanical flexible arm. This step allows en bloc retraction of the trachea and esophagus with minimal mechanical stress, thereby reducing postoperative soft tissue swelling.

**Figure 2:**
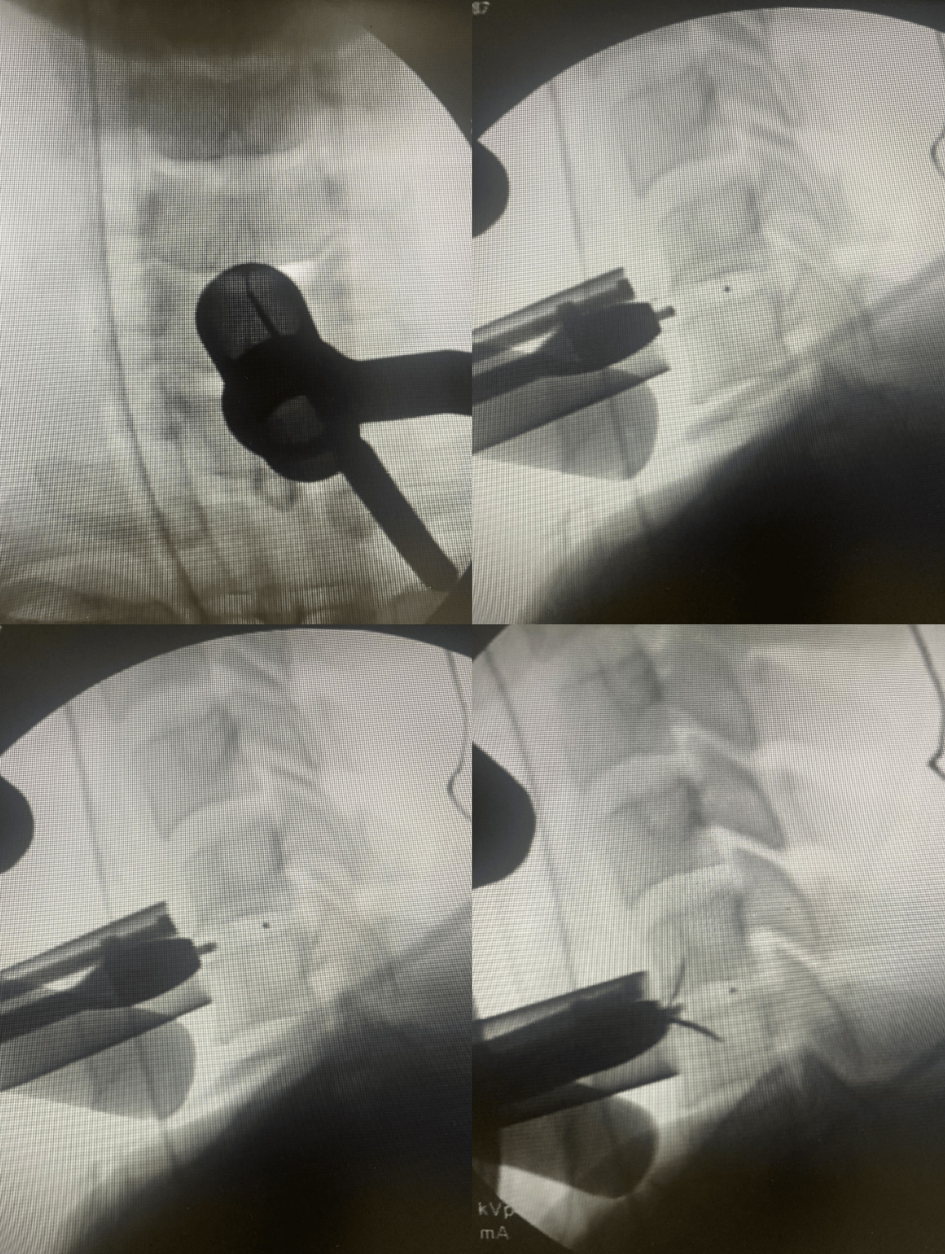
Intraoperative fluoroscopic image Fluoroscopic guidance combined with microendoscopic visualization during anterior cervical discectomy and cage insertion. Fluoroscopy ensures accurate localization of the intervertebral level and implant trajectory, while the endoscope provides direct illumination and magnification, allowing safe and precise disc removal and cage placement with reduced retraction of surrounding tissues.

**Figure 3:**
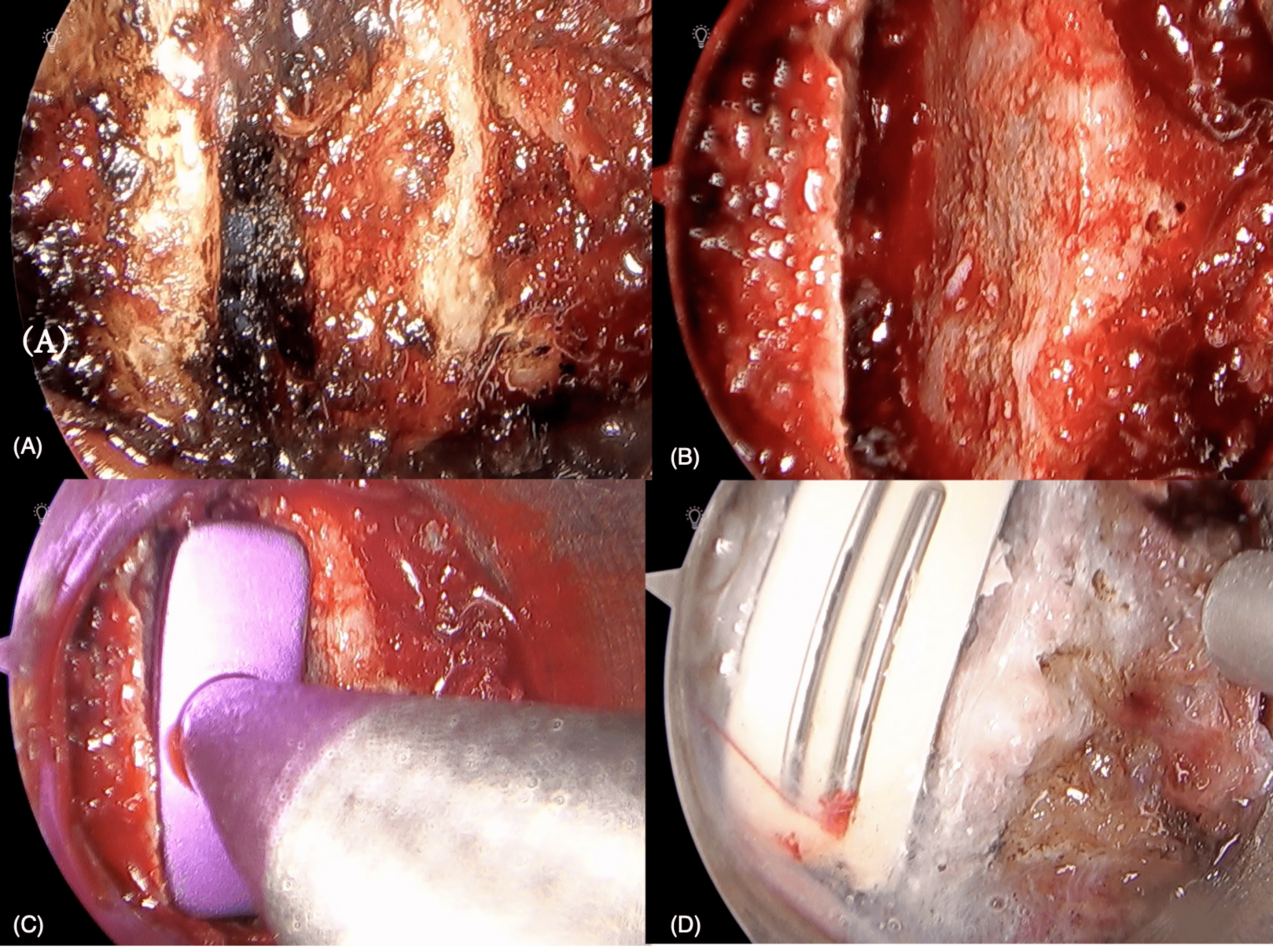
Microendoscopic view of anterior cervical discectomy and cage insertion performed under direct illumination Endoscopic visualization enables the precise resection of the disc and the clear identification of the posterior longitudinal ligament, while minimizing unnecessary retraction of surrounding tissues. Discectomy, endplate preparation, resection of anterior and posterior osteophytes, and foraminal decompression were performed using a microendoscopic technique. (A) Initial view after placement of the tubular retractor. (B) Dural sac is exposed after resection of the PLL. (C) Trial spacer inserted for cage sizing. (D) Insertion of the ROI-C^TM^ cage and self-locking blade system through the tubular retractor. This demonstrates that complete fixation can be achieved via a minimally invasive corridor, confirming the feasibility of MECAF as an alternative to conventional ACDF. PLL: posterior longitudinal ligament, MECAF: microendoscopic cervical anterior decompression and fusion, ACDF: anterior cervical discectomy and fusion

An 18-mm tubular retractor equipped with a 25-degree microendoscope (METRx endoscope system; Medtronic Sofamor Danek, Memphis, Tennessee, USA) is placed over the intervertebral disc space and stabilized with a mechanical flexible arm attached to the operating table (Figure 1). All subsequent surgical steps, including thorough discectomy, cage insertion, and fixation with a self-locking plate, are performed under direct microendoscopic visualization (Figure 2). After completion of the discectomy, the endoscopic camera is temporarily removed, and the stand-alone cervical cage (ROI-C™, Highridge Medical, Toulouse, France) is inserted through the tubular retractor (Figure 3).

Postoperative results

Patient demographics and surgical outcomes are summarized in Table 1. Between 2021 and December 2024, we performed MECAF in 20 patients. The mean patient age was 60.0 ± 15.3 years. The treated cases comprised seven cervical disc herniations, eleven cervical myelopathies, one cervical radiculopathy, and one cervical myeloradiculopathy. Notably, none of the patients experienced postoperative dyspnea, hoarseness, or dysphagia. Initially, MECAF was applied only to single-level cases (n = 14) to confirm feasibility and safety. After no significant complications were observed, the indication was subsequently expanded to include a two-level procedure, which was also completed successfully without airway-related adverse events. In addition to the absence of postoperative dyspnea, hoarseness, or dysphagia, most patients experienced improvement in their preoperative neurological symptoms, including relief of radiculopathy and functional recovery in cases of myelopathy.

**Table 1:** Patient demographics and operative data BMI: body mass index, N: number, COPD: chronic obstructive pulmonary disease

Variable	
Age at surgery (years)	60.0 ± 15.3
Female/male (N)	11/9
BMI (kg/m^2^)	23.6 ± 2.2
Operative level	
C3/4	8
C4/5	1
C4/5/6	1
C5/6	7
C6/7	3
Operative time (min)	125.4 ± 28.2
Blood loss (ml)	16 ± 18.8
Clinical pathology	
Cervical disc herniation	7
Cervical spondylotic myelopathy	11
Cervical radiculopathy	1
Cervical myeloradiculopathy	1
Comorbidity	
Hypertension, N (%)	8 (40)
Diabetes mellitus, N (%)	2 (10)
Steroid use, N (%)	2 (10)
COPD, N (%)	0 (0)
Collagen vascular disease, N (%)	3 (15)

## Discussion

Acute airway obstruction is a rare but potentially fatal complication following ACDF, underscoring the importance of measures to reduce its risk. We hypothesized that employing microendoscopic techniques might significantly mitigate this complication.

In our previous radiographic analysis, we demonstrated that postoperative swelling of anterior vertebral soft tissues was significantly reduced following MECAF compared with conventional open surgery [7]. Moreover, none of our patients experienced postoperative dyspnea, dysphagia, or hoarseness after MECAF. The incidence of postoperative dysphagia and respiratory complications in our series was notably lower compared to rates reported in the literature for open surgical approaches [8,9].

The principal causes of anterior soft tissue swelling are believed to be prolonged and forceful tissue retraction, as well as surgical manipulation. Conventional open surgery typically requires aggressive retraction of muscles, the trachea, esophagus, and neural structures to achieve adequate surgical exposure. In contrast, the tubular retractor used in MECAF significantly reduces mechanical stress on soft tissues, thereby minimizing postoperative swelling. Furthermore, endoscopic visualization enables surgeons to clearly observe deep anatomical structures, such as the posterior longitudinal ligament, under excellent illumination. A previous randomized controlled trial comparing conventional ACDF and endoscopic anterior cervical fixation (similar to our approach) reported significant reductions in postoperative pain, dysphagia, and dysphonia with the endoscopic technique; however, detailed descriptions of the surgical approach were lacking [10].

Traditionally, the Smith-Robinson and Cloward approaches, which utilize the medial border of the sternocleidomastoid muscle, have been the standard for ACDF [11-13]. However, at higher cervical levels, the sternocleidomastoid muscle migrates laterally, complicating surgical exposure. To address this challenge, we introduced an approach utilizing the lateral border of the omohyoid muscle. Because MECAF requires only a small incision, accurate preoperative assessment of the omohyoid muscle anatomy using MRI and ultrasound imaging may facilitate safe and rapid anterior cervical exposure.

Nevertheless, this technique has inherent limitations. First, since standard intervertebral distractors (e.g., Caspar pins) are not typically utilized, the procedure can be more challenging in cases of severe disc degeneration with reduced disc height, which increases the risk of endplate injury, particularly in osteoporotic patients. To overcome this limitation, we recently introduced cervical traction using the Mayfield head holder system. We employed oblique osteophyte resection at the lower edge of the superior vertebra to improve disc space exposure. Second, procedures involving upper cervical levels (C2/3 or C3/4) remain technically challenging due to the anatomical constraints posed by the mandible, which complicates optimal retractor placement and increases the risk of soft-tissue injury.

Additionally, experience with multi-level MECAF surgery remains limited. Although we recently expanded our indications to include two-level procedures, further evaluation is needed to confirm their safety and efficacy in multi-level cases. Third, as this study was designed to confirm the safety of MECAF with respect to early postoperative complications, long-term outcomes such as fusion status, recurrence, and sustained functional improvement were not evaluated. Future studies with extended follow-up will be required to address these aspects. Fourth, high BMI may represent a limitation for MECAF, particularly because excessive anterior soft tissue thickness can make tubular retractor placement and endoscopic visualization more challenging. Further study is needed to confirm the feasibility of MECAF in patients with high BMI.

## Conclusions

MECAF represents a novel application of microendoscopic surgery for cervical spine disorders, demonstrating good feasibility, safety, and encouraging short-term outcomes. Importantly, no cases of postoperative dyspnea, hoarseness, or dysphagia were observed in this initial series. However, given the small sample size and limited follow-up, these findings should be interpreted with caution. Larger comparative studies with longer-term follow-up and standardized outcome measures are warranted to more definitively establish the role of MECAF as an alternative to conventional ACDF.

## References

[1] Smith-Hammond CA, New KC, Pietrobon R, Curtis DJ, Scharver CH, Turner DA (2004). Prospective analysis of incidence and risk factors of dysphagia in spine surgery patients: comparison of anterior cervical, posterior cervical, and lumbar procedures. Spine (Phila Pa 1976).

[2] Chung WF, Liu SW, Huang LC (2020). Serious dysphagia following anterior cervical discectomy and fusion: long-term incidence in a national cohort. J Neurosurg Sci.

[3] Hardman M, Bhandarkar AR, Jarrah RM, Bydon M (2022). Predictors of airway, respiratory, and pulmonary complications following elective anterior cervical discectomy and fusion. Clin Neurol Neurosurg.

[4] Rogerson A, Aidlen J, Mason A, Pierce A, Tybor D, Salzler MJ (2021). Predictors of inpatient morbidity and mortality after 1- and 2-level anterior cervical diskectomy and fusion based on the National Inpatient Sample database from 2006 through 2010. Orthopedics.

[5] Ohba T, Akaike H, Fujita K (2021). Risk factors and assessment using an endoscopic scoring system for postoperative respiratory complications after anterior cervical decompression and fusion surgery. Spine Surg Relat Res.

[6] An SB, Lee JJ, Kim TW (2020). Upper cervical surgery, increased signal intensity of the spinal cord, and hypertension as risk factors for dyspnea after multilevel anterior cervical discectomy and fusion. Spine (Phila Pa 1976).

[7] Oda K, Nagata K, Hashizume H (2025). Efficacy and safety of microendoscopic anterior cervical decompression and fusion. Compared with conventional open surgery. J Orthop Sci.

[8] Frempong-Boadu A, Houten JK, Osborn B, Opulencia J, Kells L, Guida DD, Le Roux PD (2002). Swallowing and speech dysfunction in patients undergoing anterior cervical discectomy and fusion: a prospective, objective preoperative and postoperative assessment. J Spinal Disord Tech.

[9] DeBehnke DJ, Havel CJ (1994). Utility of prevertebral soft tissue measurements in identifying patients with cervical spine fractures. Ann Emerg Med.

[10] Soliman HM (2013). Cervical microendoscopic discectomy and fusion: does it affect the postoperative course and the complication rate? A blinded randomized controlled trial. Spine (Phila Pa 1976).

[11] Jenis LG, Leclair WJ (1994). Late vascular complication with anterior cervical discectomy and fusion. Spine (Phila Pa 1976).

[12] Arumalla K, Bansal H, Jadeja J (2021). Anterior approach to the cervical spine: elegance lies in its simplicity. Asian J Neurosurg.

[13] Cho W, Buchowski JM, Park Y, Maeda T, Nabb CE, Riew KD (2012). Surgical approach to the cervicothoracic junction: can a standard Smith-Robinson approach be utilized?. J Spinal Disord Tech.

